# Feasibility of an amazonian dance protocol for people with Parkinson’s disease

**DOI:** 10.1016/j.isci.2025.114396

**Published:** 2025-12-10

**Authors:** Carlos Cristiano Espedito Guzzo Junior, Raquel Arigony Corrêa Sant`anna Prates, Thayara Maize da Silva Tabayara, Luma Sabrina da Silva Moraes, Maria Vitoria Andreazza Duarte, Elren Passos-Monteiro, Aline Nogueira Haas

**Affiliations:** 1 Department of Physical Education, Physiotherapy and Dance, Federal University of Rio Grande do Sul, Rua Felizardo, 750, Jardim Botânico, Porto Alegre CEP: 90690-200, Rio Grande do Sul, Brazil; 2 Departament of Sports, School of Physical Education, Campus III, State University of Pará, Avenida João Paulo II, 817, Marco, Belém CEP: 66095-490, Pará, Brazil; 3 Institute of Health Sciences, Graduate Program in Human Movement Sciences, Federal University of Pará, Avenida Generalíssimo Deodoro, 01, Umarizal, Belém CEP: 66050-160, Pará, Brazil; 4 Global Brain Health Institute, Trinity College Dublin, Dublin 2, Ireland

**Keywords:** Public health, Neuroscience

## Abstract

Dance is a promising non-pharmacological intervention for people with Parkinson’s disease (PwP), enhancing mobility, balance, coordination, and cognition. However, the feasibility and safety of Amazonian dances have not yet been evaluated. This study assessed the feasibility and acceptability of the Amazonian Dance Protocol (ADp) for PwP in two Brazilian regions, North and South. Over 12 weeks, 24 PwP attended ADp sessions twice weekly. Feasibility outcomes included recruitment, adherence, acceptability, and safety, assessed through questionnaires and interviews. The study reached an 89% enrollment rate, with high adherence (93.75%) and a low dropout rate (16.6%). Satisfaction levels were high (mean: 4.5/5), and 94% of participants would recommend the program. Reported benefits included improvements in motor function, emotional well-being, and social engagement, despite some coordination and balance challenges. One fall occurred, unrelated to the intervention. The ADp demonstrated strong feasibility and acceptance, supporting further research on its long-term effects and broader implementation.

## Highlights

• First study testing Amazonian dances for people with Parkinson’s
• Amazonian Dance Protocol is feasible and highly accepted by participants
• Culturally rooted approach expanding neurorehabilitation strategies
• Traditional dances as accessible tools for brain health promotion

## Graphical abstract

**Figure:**
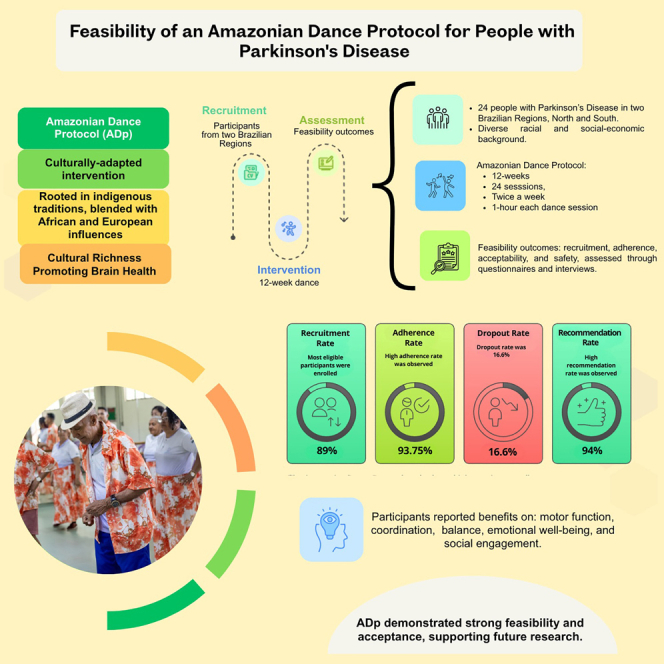

## Introduction

Despite advances in the treatment of Parkinson’s disease (PD), the high incidence of the disease continues to require specialized care and ongoing monitoring to improve patients' quality of life and well-being. Existing treatments focus on symptom management, slowing disease progression, maintaining autonomy and functional independence, and improving patients' quality of life.^1^^,^^2^^,^^3^ Among non-pharmacological interventions, dance has emerged as a safe and effective complementary approach, enhancing people with PD (PwP) mobility, flexibility, balance, and coordination.^4^

Dance distinguishes itself from other non-pharmacological therapies for PD due to its multidimensional nature, encompassing physical, emotional, and social aspects.^5^ This integration allows PwP to address their symptoms in a holistic manner while enjoying a practice that involves rhythmic, music, and movement, making it more engaging and enjoyable. Furthermore, dance stimulates cognitive functions, improving executive function, attention, and memory.^6^ The social and emotional components of dance are equally noteworthy, fostering interaction, reducing social isolation, and improving emotional well-being.^7^ By offering a form of artistic and creative expression, dance encourages self-expression, which can increase adherence and sustained engagement, contributing to a better quality of life.^8^^,^^9^

Systematic reviews corroborate the benefits of dance, particularly in functional mobility and motor symptoms PwP.^4^^,^^10^^,^^11^ However, there is still a gap in the literature regarding the effects of dance on non-motor symptoms^1^^,^^2^^,^^7^^,^^11^ and the impacts of different dance genres.^3^^,^^12^^,^^13^^,^^14^^,^^15^ While some studies have explored the benefits of Brazilian dance, such as Samba and Forró, on motor symptoms,^3^^,^^14^^,^^16^ there is limited evidence on the effects of other Brazilian dances genres, such as Amazonian dances, on non-motor and motor symptoms. Current dance interventions for PwP, while effective, often fail to reach diverse populations due to limitations in accessibility, cultural relevance, and program adaptability. Introducing alternative dance genres, such as Amazonian Dances, can address this gap by providing a culturally meaningful and accessible non-pharmacological intervention.

The Amazonian dances are an example of cultural expression among the many popular regional dances of Brazil. They possess a distinct aesthetic identity, characterized by specific movement patterns such as pronounced hip movements, knee flexion, arched arms, improvisation, among others.^17^ These dances are part of Brazil’s cultural heritage and are predominantly practiced in the Brazilian northern region. They are rooted in indigenous traditions, blended with African and European influences, and their cultural strength lies in their deep connection with nature.^18^

Thus, the purpose of this study is to evaluate the feasibility and acceptability of the Amazonian dance protocol (ADp) in PwP in two distinct Brazilian regions (North and South), exploring the cultural adaptation of a Brazilian dance traditionally linked to the Brazilian North Region. These regions represent two culturally and geographically contrasting contexts, which allows for an examination of how local traditions, social environments, and regional characteristics may influence the implementation of a culturally adapted non-pharmacological intervention. A culturally adapted intervention is essential to ensure relevance and engagement.

Conducting a feasibility study is a critical step before implementing a randomized controlled trial (RCT), as it enables the evaluation of the intervention’s applicability, safety, economic viability, and participant adherence, particularly in different regions of the same country that present diverse cultural, social, and environmental characteristics.^19^ Additionally, it provides insight into participant acceptability and engagement, helping to refine the study design and optimize the intervention before full-scale implementation.^19^

To the best of the author’s knowledge, this is the first study to systematically evaluate the feasibility and effectiveness of an ADp for PwP. Over 12 weeks, participants experienced the ADp, a cultural manifestation, which, among the various popular regional dances in Brazil, is characterized by distinctive aesthetic features such as pronounced hip movements, knee flexion, arched arms, improvisation, and other specific elements.^20^

## Results

### Participants’ demographic characteristics

Table 1 shows the participants’ demographic and clinical characteristics. Twenty-four PwP were enrolled in the study, of whom 16 completed all stages of the intervention (ADN = 8; ADS = 8).

**Table 1:** Participants’ demographic and clinical characteristics

Variable	(ADN) mean ± SD n (%)	(ADS) mean ± SD n (%)
MDS-UPDRSIII	49.88 ± 3.65	39.38 ± 4.02
H&Y	2.69 ± 0.46	1.75 ± 0.71

**Sex**		

Female	2 (25%)	5 (63%)
Male	6 (75%)	3 (37%)

**Age**		

60–69 years	2 (25%)	2 (25%)
70–79 years	6 (75%)	6 (75%)

**Race**		

White	1 (12.5%)	7 (88%)
Black	1 (12.5%)	1 (12%)
Indigenous/Mixed race	6 (75%)	–

**Marital Status**		

Single	2 (25%)	–
Married	3 (38%)	6 (75%)
Cohabiting (living with partner)	2 (25%)	–
Widowed	1 (12%)	2 (25%)

**Education level**		

Primary school	5 (63%)	–
Secondary school	1 (12%)	1 (12%)
Higher Education	2 (25%)	3 (38%)
Postgraduate	–	4 (50%)

**Dance Practice Time**		

Less than 1 year	6 (75%)	8 (100%)
More than 10 years	2 (25%)	–

**Dance Experience**		

Not at all	2 (25%)	7 (87.5%)
Beginner	–	1 (12.5%)
Intermediate	4 (50%)	–
Advanced	1 (12,5%)	–
Professional	1 (12,5%)	–

**PD diagnosis**		

0-2 years	1 (12%)	3 (38%)
3-5 years	4 (50%)	2 (25%)
6-10 years	2 (25%)	2 (25%)
11 years or more	1 (12%)	1 (12%)

**Prolopa**		

1 tablet 2 times a day	1 (12.5%)	–
1 tablet 3 times a day	2 (25%)	2 (25%)
1 tablet 4 times a day	4 (50%)	3 (38%)
1 tablet 6 times a day	1 (12.5%)	1 (12%)
1 tablet 8 times a day	–	1 (12.5%)
2 tablets 6 times a day	–	1 (12.5%)

**Cognition**		

CU	1 (12.5%)	1 (12.5%)
MCI-range	7 (87.5%)	7 (87.5%)

**Risk of Fall**		

Low risk	1 (12.5%)	–
Moderate risk	4 (50%)	2 (25%)
High risk	3 (37.5%)	6 (75%)

Note: SD - Standard Deviation; n - number of participants; % - percentage.

Regarding sex, the ADN group is predominantly composed of males (75%), while the ADS group has a female majority (62%). This may reflect local cultural and participation patterns in the Northern region of Brazil.

Concerning age, both groups show a higher concentration of participants in the 70–79 age range (75%), with lower representation of individuals aged 60–69 (25%). In terms of race, greater diversity is observed in the ADN group, which consists of 75% Indigenous or mixed-race, 12.5% White, and 12.5% Black. Conversely, the ADS group is predominantly composed of White people (88%), with only 12% Black, and no Indigenous or mixed-race.

In the ADN group, there is greater variation of marital status: 25% are single, 38% are married, 25% are cohabiting, and 12% are widowed. In the ADS group, the majority are married (75%), and 25% widowed, with no single or cohabiting participants in this group.

In terms of education level, the ADN group primarily consists of people with primary education (63%), whereas the ADS group shows a higher level of education and more balanced distribution, with 50% having secondary or higher education and 50% holding postgraduate qualifications.

The disease duration reveals a distinct distribution between the groups. In the ADN group, half of the participants (50%) have been diagnosed for 3–5 years, followed by 25% for 6–10 years. In the ADS group, the distribution is more uniform, with 25% in each time range (3–5 years and 6–10 years).

The use of Prolopa varies between the groups. In the ADN group, the most common dosage is 1 tablet, 4 times a day (50%), followed by 1 tablet, 3 times a day (25%), and 1 tablet, 2 or 6 times a day (12.5% each). In the ADS group, the dosage is more diverse, with 38% of the participants using 1 tablet, 4 times a day, 25% using 1 tablet, 2 times a day, and the remaining participants distributed among higher doses, including 1 tablet, 8 times a day (12.5%).

According to MoCA cutoff scores adapted to the Brazilian population,^21^ most of the participants in both groups were categorized as *MCI-range*, with a single exception in each group (ADN and ADS), where one participant was categorized as CU.

In the ADN group, most participants are classified as *Moderate Risk (50%) or High Risk (37.5%)*, which suggests a significant probability of falls occurring in this group, with only one person in *Low Risk*. In the ADS group, *High Risk* predominates with 75%, suggesting a higher perception of risk and fear of falls in this group. The absence of participants in *Low Risk* is an important data point, highlighting that all members of this group present some degree of risk related to falls.

In terms of Dance Practice Time, there is a noticeable contrast between the ADN and ADS groups. In the ADN group, most participants (75%) have been practicing dance for less than one year, while 25% have more than 10 years of experience. This suggests a mix of relatively new dancers alongside those with substantial practice history. On the other hand, in the ADS group, all 8 participants (100%) have been practicing dance for less than one year. This indicates a more homogenous group in terms of dance experience, with all members relatively new to the practice.

When it comes to Dance Experience Classification, there is also a distinction between the two groups. In the ADN group, the classification is more diverse. 25% of the participants consider themselves to have no experience at all, while 25% identify as Intermediate, 50% as Advanced, and 12.5% as Professional. This variety reflects a wide range of dance backgrounds, from complete beginners to those who have reached advanced levels of expertise.

In contrast, the ADS group predominantly classifies themselves as “Not at all” experienced, with 87.5% falling into this category. Only one participant (12.5%) considers themselves as a “Beginner.” This suggests that the ADS group has a less varied range of self-assessed dance experience levels, with most participants not seeing themselves as having much dance background.

### Feasibility and acceptability

**Figure 1:**
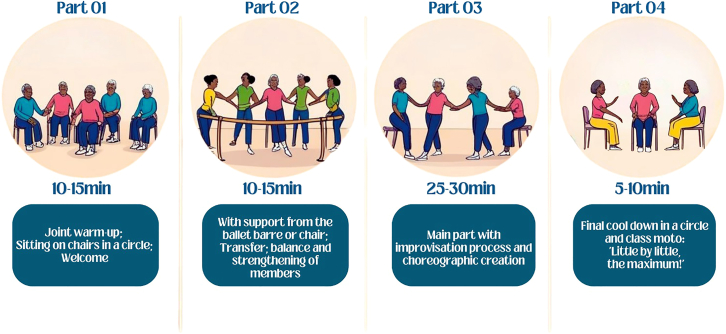
Amazonian dance protocol structure

The feasibility and acceptability of the ADp (Figure 1) criteria were met or exceeded, as described in Table 2, which summarizes the established benchmarks, target goals, and corresponding data. Figure 1 illustrates the structure of the ADp sessions.

**Table 2:** Feasibility and acceptability benchmark goals and evolution

Data of interest and Measures	Benchmark goals for Success	Results	Success
**Feasibility of Recruitment and enrollment:** (1) enrollment rate; (2) recruitment source; (3) reason for non-eligibility; (4) reasons for withdrawal of interest	(1) 80% enrollment rate; (2) NCS; (3) NCS; (4) NCS	1. 89% 2. Social media (81.51%); referred by others (5.10%); study leaflet (2.91%); other (10.48%) 3. Individuals with other neurological or chronic diseases (39.25%); ≥80 years old (2.49%); individuals unable to ambulate without assistance (0.58%); mental health concerns (3.16%); discomfort with the protocol (5.79%); did not complete participation or missed some evaluations (33.14%). 4. Medical emergencies (3.85%); lack of follow-up (1.05%); unknown reasons (1.74%).	(1): Yes; (2), (3), (4): N/A
**Retention:** dropout rate	≤16.66% (*n* = 4) dropout ADN ≤16.66% (*n* = 4) dropout ADS	16.66% (*n* = 4) dropout ADN (received an additional diagnosis besides PD; did not participate in the post-intervention; had trouble adapting to the protocol). 16.66% (*n* = 4) dropout ADS (due to health issues; chose another form of physical activity).	Yes
**Treatment adherence**: (1) number of sessions achieved; (2) number of daily practices; (3) reason for non-adherence	(1) ≥ 75% (*n* = 15) completing >24 sessions; (2) > 2 days/week; (3) NCS	1. 93.75% (*n* = 15) completed, 24 sessions. 2. Average of 2 days/week (SD = 1.717) 3. Illness and doctor’s appointments	(1) Yes; (2) Yes; (3) N/A
Safety	One adverse event	An adverse event was reported involving a participant who experienced malaise and dizziness, leading to a loss of balance and a fall during an activity. The fall was not caused by the activity but was attributed to a pre-existing condition, as noted by the caregiver.	Yes
**Treatment** (1) 5-point Likert; (2) interview	(1) Average score ≥4.5; (2) NCS	1. Mean = 4.5 (SD = 0.62) 2. Participants considered the intervention safe, enjoyable, and beneficial, expressing satisfaction with its content, method of delivery, frequency, and duration.	(1) Yes; (2) Yes
Credibility and Expectation	High acceptance and appreciation of the protocol in both regions.	Satisfaction rate of ≥90%	Yes
Continuation and recommendation	89,9% interested in continuing, 94% would recommend the intervention	94% (15/16 participants) expressed interest in continuing and recommended the program	Yes

Note: NCS: no criteria set; N/A: not applicable; SD = Standard Deviation.

### Recruitment and enrollment

Of the 42 participants who underwent the screening interview, 36 (96%) were deemed eligible to participate in ADp. However, along the way, three withdrew due to scheduling conflicts, two faced difficulties accessing the session location, and two were excluded for engaging in other simultaneous activities. Ultimately, 29 participants met the eligibility criteria. However, between screening and the start of the study, changes in health conditions or personal circumstances led some eligible participants to withdraw, leaving 24 (89%) who were successfully enrolled. A total of 12 participants were assigned to each group.

### Amazonian Dance Protocol retention and adherence

A total of 24 participants were enrolled, with 12 in each group. However, in the ADN group, four participants discontinued the intervention for different reasons: one received an additional diagnosis besides PD (*n* = 1), one did not participate in the post-intervention assessment (*n* = 1), and two experienced difficulties adapting to the protocol (*n* = 2). Similarly, in the ADS group, four participants withdrew from the study: three due to health issues (*n* = 3) and one opted for another form of physical activity (*n* = 1).

Sixteen participants completed the study, with 15 (93.75%) achieving at least 75% adherence to the intervention, meeting the established goal. On average, participants practiced 2 days per week (SD = 1.73, range: 1–7 days), corresponding to approximately 84% of the planned duration (24 days) (Table 2).

### Treatment credibility and expectation

At the end of the ADp, the participants (*n* = 16) completed a feasibility questionnaire. Across both groups (North and South), the participants assigned an average satisfaction score of 4.5 (SD = 0.62) (Table 2). This corresponds to an average satisfaction rate of 90% relative to the maximum score on the scale, indicating high acceptance and appreciation of the protocol in both regions.

### General perception of the Amazonian Dance Protocol benefits on Parkinson’s disease symptoms

The participants showed a highly positive perception of the potential impacts of the ADp on PD symptoms. Most of the participants rated the intervention as highly beneficial, with responses concentrated at the upper levels of the scale (4 and 5). When asked about the benefits on non-motor symptoms (such as memory and language) and motor symptoms (such as walking, rigidity, and slowness), the mean scores were 5 ± 0.49 and 5 ± 0.50, respectively.

### Satisfaction and acceptability of the Amazonian Dance Protocol

The participants reported high levels of satisfaction and acceptability regarding the three assessed dimensions: Well-being and joy (5 ± 0.61); Comfort during sessions (5 ± 0.62); and Motivation to participate (5 ± 0.48). These results reinforced the ADp acceptance as a feasible and enjoyable non-pharmacological intervention for PwP.

Despite the overall high ratings, only two participants - one from the ADN and one from the ADS - assigned a score of 3 to the “comfort during sessions” dimension, indicating a moderate perception in this aspect. This exception in “comfort ratings during the class” underscores the importance of continually modifying and adapting the ADp activities to each participant, respecting their individualities.

### Fear of falling during the activities

The overall average score for fear of falling during the activities was 2.5 ± 1.26, indicating a low level of concern. However, the distribution of responses revealed some variations: two participants reported a moderate level of concern (score 3), while five participants expressed higher levels of concern (score 4). These findings suggest that, while the overall average indicates low concern, some participants experienced moderate to high levels of fear of falling.

Additionally, it is important to highlight that participants with higher fear of falling scores on the Fall Efficacy Scale (FES) (Table 1) also reported greater concern about falling during the intervention. However, it remains unclear whether this fear was specifically triggered by certain movements, such as turns and balance exercises, or if it was inherently linked to Parkinson’s disease itself. This ambiguity underscores the need for continuous monitoring of fear of falling, as fluctuations in perceived safety can negatively impact both confidence and performance. Therefore, implementing enhanced safety measures, psychological support, and targeted training strategies is essential to mitigate fear and improve participants’ overall confidence during the activities.

### Dance studio and instructor qualifications

The dance studio received a high level of approval (5 ± 0.55). Only one participant from the ADN was assigned a score of 3, indicating a neutral perception. Similarly, the instructors’ qualifications received high ratings (5 ± 0.53). Again, only one participant assigned a score of 3, while all others expressed high levels of satisfaction about the instructors' expertise. These results underscore the importance of the environment and team in the success of the ADp. The high satisfaction about both, dance studio and instructors' qualifications, reinforces the quality of the program’s conditions and their positive impact on the participants’ overall experience.

### Performance of the Amazonian Dance Protocol activities

The results about the ease or difficulty of performing the ADp demonstrate variability in participants’ perceptions. While most found the dance movements to be easy and accessible, others experienced challenges, particularly with movements requiring greater motor coordination and rhythmic synchronization. The wide range of scores underscores the importance of tailoring activities to individual needs and providing appropriate support for participants facing greater difficulties.

Most of the participants felt it was easy to perform the movement of flexing and extending the spine (mean score 3.5 ± 1.77). However, one participant assigned higher scores (up to 7), suggesting that this type of movement can be difficult to perform.

The improvisation proposals were also considered relatively easy (mean score 3.5 ± 2.39). However, responses varied, as some participants gave scores of 8 or 9, indicating more difficulty in adapting to improvisation, especially for those with motor and cognitive limitations.

The turning and rotational movement were considered with moderate difficulty (mean score 4.0 ± 2.18). Nevertheless, four participants gave scores as high as 9, indicating challenges for some of them in performing this movement.

The stepping to the lateral, diagonal, or in circles movements were also considered with moderate difficulty (mean score 4.5 ± 2.38). The assignment of higher scores (up to 8) by some participants to the circular step movements suggests that these movements were particularly challenging for them.

The same happened with the rhythmic stepping pattern, which was considered moderate difficulty (mean score 4.0 ± 2.30). Some participants rated this as more difficult (scores up to 9), reflecting individual challenges with synchronization to the dance rhythm.

The coordinating arms and legs was the most challenging movement according to the participants perceptions (mean score 5.0 ± 2.59). The wide range of responses, including scores of 10 (one participant), 9 (one participant), and 8 (two participants), underscores the motor coordination difficulties experienced by most participants.

### Adverse event

During the study, an adverse event was reported involving a participant who experienced dizziness and subsequently lost balance, resulting in a fall while performing an activity standing behind a chair. Notably, the participant had a history of recurrent dizziness in daily life, as previously reported by their caregiver, and the fall was attributed to this pre-existing condition rather than the activity of the ADp itself.

The situation was promptly managed, as a dance instructor/physiotherapist was nearby and provided immediate assistance. The fall did not result in serious injuries; the participant did not hit the head or lose consciousness and was assessed and reassured at the time of the incident. This event underscores the importance of accounting for participants’ pre-existing clinical conditions and implementing robust safety measures, such as having trained instructors during the activities to ensure swift and effective interventions when needed.

Following the incident, the participant was advised to pay close attention to bodily sensations, particularly dizziness, and to avoid sudden movements when sitting, standing, or turning to reduce the risk of falls. Additionally, the participant was encouraged to remain seated on days when dizziness persisted. Due to the continued recurrence of dizziness in daily life, the participant ultimately had to discontinue their participation in the intervention to focus on diagnosing the underlying cause of the issue and adjusting the medication regimen.

### Qualitative results

Sixteen participants who completed the ADp were interviewed at the end of the intervention. Key themes emerged from the analysis: group participating experience, psychological effects of the ADp, and satisfaction with the ADp. These themes reflect the participants’ diverse experiences and the multifaceted impact of the intervention.

#### The participants’ engagement experience

The group participating experience was particularly valuable in alleviating the loneliness experienced by participants in their daily life. Approximately half of the participants mentioned that ADp provided a unique opportunity to connect safely with others, especially with people facing similar challenges related to PD, enabling the exchange of experiences.

Most of the participants reported a strong sense of “community,” describing their peers as a “team” or “friends.” One participant noted: *“We all felt welcomed and loved, and we shared what we were going through with people we had never met before … we became friends.”* (#ZN). The participants expressed comfort in sharing aspects of their personal lives and appreciated the group support: *“It was about connecting with the people here. I enjoy being around the teachers and friends; they are very cheerful.”* (#CN).

For one participant, the program served as a bridge between isolation and reintegration into in-person social environments: *“In the classes, everyone works hard to welcome* each *other, especially the teachers. The most important thing I learned here was being with people who have the same disease. Everyone brings their own challenges, which may be greater or smaller than mine, but we are together to understand and support each other. It’s a constant learning process. I wake up motivated to come to the classes.”* (#DS).

Half of the participants highlighted that the ADp was as an effective form of communication and socialization for those who typically considered themselves introverted. Enjoying the ADp created a safe environment for individuals initially hesitant to participate due to fears of not performing movements correctly. One participant said: *“I learned that I could exercise while respecting my limitations—something I thought I couldn’t do before. When I started, I was afraid I wouldn’t keep up, but the teachers were very attentive and adapted everything for me.”* (#MR).

The group participating experience also underscores the importance of socialization and mutual support in the rehabilitation process and improvement of participants’ quality of life. It highlights the vital role of group dynamics in encouraging adherence and sustained participation in the program.

#### Psychological effects of the Amazonian Dance Protocol

The participants reported various psychological benefits and improvements in their overall health and well-being, directly attributed to practicing the ADp. Most of the participants (70%) observed an increase in body awareness, fostering a deeper mind-body connection. One participant shared: *“It was interesting to see how certain movements are adapted and used in dances that, at first glance, seem to have nothing in common. I’m trying to move much more, and I’m also trying to think about my movements.”* (#CF). Another participant stated: *“My body is doing so much better, and it was great that I signed up. I wasn’t doing well before, but now I can exercise, I’ve gone back to reading, listening to music, watching shows, and even keeping up with my wife better.”* (#JT).

The experience with the ADp not only enhanced participants' body perception but also encouraged them to value their capabilities and the everyday aspects of life. 64% of the participants observed significant improvements in the quality and range of their movements, describing it as a process of rediscovering their physical capabilities. One participant stated: *“I wouldn’t have discovered this if I hadn’t joined this class. Now I can move my arm behind my head … I call that progress and growth.”* (#DP). This observation underscores not only physical improvements, but also the positive psychological impact of regaining the ability to perform previously challenging movements.

Another noteworthy aspect was the impact of the practice on mindfulness, a central element of the protocol that helped participants better manage their emotions. Several participants mentioned how dance helped them release negative emotions and find a space of emotional balance. As one participant expressed: *“As bad as the disease is, even though it isn’t reversible, we can control it so that it doesn’t become so aggressive.”* (#JA). This statement highlights how regular engagement in body-focused activities can aid in emotional regulation and contribute to mental health resilience.

Overall, the ADp not only facilitated physical improvement but also played a vital role in promoting mind-body connections, enabling participants to reframe their self-perception, capabilities, and emotions. The experience offered a new understanding of their own limits and possibilities while strengthening their connection to culture and identity.

#### Levels of satisfaction with the Amazonian Dance Protocol

The participants expressed high levels of satisfaction with the ADP, highlighting key aspects such as well-being, comfort during the sessions, and motivation to participate. Many reported feeling happy and more energized after the activities, stating that the sessions provided a sense of well-being and enjoyment. Some mentioned feeling lighter and more relaxed, as if the ADp had helped relieve tension in both body and mind. Regarding comfort, participants emphasized the suitability of the activities, commenting that they felt welcomed and respected in their limitations, which facilitated active participation: “*The classes were tailored to my needs, and I felt comfortable the whole time*” (#MR).

Motivation to continue participating was also a recurring theme, with many expressing enthusiasm for each session: “*I felt motivated to come back every week, not only because of the activities but also because of the positive environment and the support from the instructors*” (#ES). This statement reinforces the acceptance of the ADp as a feasible and enjoyable intervention, contributing to the quality of life and engagement of PwP.

## Discussion

The aim of this study was to evaluate the feasibility and acceptability of the ADP in PwP in two Brazilian regions (North and South). The ADP was considered feasible and widely accepted, as evidenced by high levels of satisfaction and engagement among the participants from the two Brazilian regions.

The ADp demonstrated a high acceptability among the participants, from both Brazilian regions (North and South), reflecting high levels of satisfaction with the proposed activities. Lima et al.^22^emphasize that interventions with strong cultural relevance tend to foster greater engagement, leading to better clinical outcomes. Similarly, Bek et al.^15^ found that dance-based programs yielded superior adherence compared to traditional exercise programs, due to their emotional and social appeal. These findings align with this study results, highlighting that culturally adapted interventions can significantly enhance participant motivation and adherence.

These findings reinforce the importance of culturally adapted interventions that are co-developed with the participation of the participants, ensuring that interventions meet the specific needs of the target population. Despite this, Marquez et al.^23^ suggest that interventions incorporating structured feedback and psychological support may further optimize satisfaction and long-term engagement. Integrating these elements into future iterations in future dance protocols could enhance their effectiveness and expand their reach.

The cultural context plays a significant role in the effectiveness of interventions for PwP, as locally adapted practices can enhance participants' motivation and engagement. The ADp incorporates elements of Amazonian dance, respecting Brazilian cultural traditions and fostering a sense of belonging and identity among the participants. This aligns with the findings of Tilmann et al.,^24^ Solla et al.,^25^ and Elpidoforou et al.^26^ who observed that culturally adapted interventions often produce positive impacts across diverse populations. For instance, Solla et al.^25^ demonstrated that Sardinian folk dance improved cognitive and motor functions in PwP, while Elpidoforou et al.^26^ highlighted that integrating cultural elements amplified the emotional and social significance of rehabilitation activities. Unlike these protocols, the ADp is rooted in Amazonian cultural heritage, incorporating region-specific rhythms, movements, and symbolic elements, offering a distinct sensory and social experience for PwP.

Dance, as a form of cultural expression, can serve as an innovative way to alleviate PD symptoms, emphasizing the importance of cultural aspects in the design of non-pharmacological interventions for PwP. Additionally, Pinto et al.^27^ emphasize that tailoring interventions to specific cultural and environmental contexts not only increases adherence but also enhances the overall effectiveness of the program. These findings underscore the value of culturally adapted interventions, such as the ADp, in addressing the diverse needs of PwP and ensuring meaningful engagement.

The potential of the ADp to promote social integration and emotional well-being was perceived by the participants. Tilmann et al.^24^ emphasize that the impact of dance on emotional variables often depends on the degree of social and emotional support integrated into the intervention. This aligns with Solla et al.,^25^ who suggested that adding psychosocial components to dance protocols can mitigate negative emotional outcomes. Pinto et al.^27^ also highlight the value of combining physical activity with psychosocial interventions to address emotional variables comprehensively.

The collected reports highlight that emotional and psychological aspects, alongside motor and non-motor benefits, play a key role in adherence and intervention effectiveness. This aligns with the findings of Delabary et al.,^28^ who observed that the effects of dance on non-motor symptoms, including quality of life, are often subtle and require multidimensional approaches for meaningful changes. This reinforces the idea that interventions such as dance, for people with neurodegenerative diseases, can be most effective when they consider not only physical aspects but also emotional, cognitive, and social dimensions. The high adherence rate suggests the intervention was well received, supported by a motivating environment, socialization, and perceived benefits.

This study presents several strengths and innovative aspects. To our knowledge, this is the first study to explore the feasibility and acceptability of the Amazonian dances as an intervention for PwP. By incorporating a culturally relevant and regionally rooted practice, this research expands the repertoire of non-pharmacological approaches in neurorehabilitation and highlights the potential of traditional dances as accessible and engaging tools for physical and cognitive stimulation. The structured feasibility framework, adherence guidelines, and inclusion of multidimensional outcomes reinforce the methodological robustness of the study.

Future studies could investigate how the ADp can be culturally adapted to diverse settings beyond Brazil, assessing its scalability and effectiveness in meeting the specific needs of PwP across different regions and countries. Larger, controlled studies are warranted to draw more definitive conclusions about the effects of this complementary non-pharmacological intervention on PD. A future clinical trial is planned, which will incorporate pre- and post-intervention clinical assessments to provide a more comprehensive evaluation of the Amazonian Dance Protocol’s potential effects. In addition, implementation studies could explore strategies for scaling and integrating this culturally adapted intervention into public rehabilitation programs, incorporating co-design with community members to better understand its impact. Such studies may also systematically examine the role of caregiver involvement in dance-based interventions for PD, as they play an important role in providing both emotional and logistical support.

### Limitations of the study

Some limitations of the study should be considered. Some participants faced commuting difficulties, emphasizing the need for better accessibility in future interventions. These difficulties may be related to individual variations in the disease progression, emphasizing the need for personalized adaptations. Future research can explore the relationship between movement hesitancy, protocol effects, and Parkinson’s-related balance impairments.

## Resource availability

### Lead contact

Further information and requests for resources and materials should be directed to and will be fulfilled by the Lead Contact, Aline Nogueira Haas (email: aline.haas@gbhi.org).

### Materials availability

This study did not generate new unique reagents or materials.

### Data and code availability

All data reported in this article will be shared by the lead contact upon reasonable request.

No custom code or software was generated or used in this study.

Any additional information required to reanalyze the data reported in this article is available from the lead contact upon request.

## Acknowledgments

We gratefully acknowledge the participants who contributed to this project and the professors who served as research assistants in this study. This research was supported by the Alzheimer’s Association in partnership with the Global Brain Health Institute (GBHI) under grant application 10.13039/100015442GBHI ALZ UK-24-1068625 and by the Coordenação de Aperfeiçoamento de Pessoal de Nível Superior – Brasil (10.13039/501100002322CAPES) – Finance Code 001.

## Author contributions

C.C.E.G.J.: conceptualization, investigation, visualization, writing – original draft, writing—review and editing; R.A.C.S.P.: investigation, writing – original draft; T.M.S.T, M.V.A.D., and L.S.S.M.: resources, writing; E.P.M.: conceptualization, supervision, and writing – review; A.N.H.: conceptualization, funding acquisition, supervision, and writing – review and editing. All co-authors have read and approved the final version of the article.

## Declaration of interests

The authors declare no conflict of interest.

## STAR★Methods

### Key resources table

**Table:** 

REAGENT or RESOURCE	SOURCE	IDENTIFIER
**Deposited data**		

Data for the Amazonian Dance Protocol for people with parkinson’s disease	https://data.mendeley.com/datasets/3wgxpsbch7/1	https://doi.org/10.17632/3wgxpsbch7.1

**Software and algorithms**		

Microsoft Excel	Microsoft Corporation	N/A
Adobe Illustrator 2020	Adobe	N/A

**Other**		

Human participants	People with Parkinson’s disease (PwP)	Diagnosed by neurologist, H&Y I–III
Unified Parkinson’s Disease Rating Scale (UPDRS)	Movement Disorder Society	https://www.movementdisorders.org

### Experimental model and study participant details

People diagnosed with idiopathic PD were recruited from Dance Community Projects, Parkinson’s Associations, and HCPA, via social media, flyers, and an electronic newsletter. Inclusion criteria were: (a) standard clinical diagnostic criteria determined by a neurologist (Postuma et al., 2015), (b) both sexes and social-economically and racially diverse. The participants were eligible if they were: aged 60–80 years; Hoehn and Yahr (H&Y) stages I–III; ≥1 year undergoing medical treatment for PD with regular use of anti-parkinsonian drugs; and able to understand the verbal instructions for the tests and walk with no walking aid. Exclusion criteria were: (a) those with other associated neurological diseases or chronic diseases, (b) having recently undergone surgery, and/or received deep brain stimulation in at last 3 months; (c) not completing the training and/or not participating in the assessments.

The sample size was determined based on practical and logistical considerations within the recruitment period, in line with other feasibility studies in this field.^9^^,^^24^^,^^26^ After being recruited, participants underwent a screening interview to determine if they met the eligibility criteria for the study. The study was conducted in two distinct regions of Brazil (North and South), and the participants were divided into two groups: Amazonian Dance North Region (ADN), strongly identified with local practices; and Amazonian Dance South Region (ADS), where such identification is less present.

The choice of the North and South regions of Brazil was based on cultural, logistical, and representativeness factors. The Brazilian North Region, with its strong influence from Amazonian manifestations, and the South, with distinct cultural influences, allow for an assessment of the protocol’s adaptation in different cultural contexts. Additionally, the feasibility of conducting research in these locations was enhanced by specialized centers for Parkinson’s Disease treatment and the availability of qualified researchers. The inclusion of these two regions also enables an initial analysis of the protocol’s acceptance in contrasting socioeconomic realities, providing essential data for the future expansion of the study.

The study adhered to ethical guidelines and received approval from the Hospital de Clínicas de Porto Alegre (Porto Alegre Clinics Hospital/HCPA) (CAAE: 75675023.9.0000.5327) and Federal University of Pará Ethics Committee (CAAE: 75675023.9.3002.0018). All the participants provided written consent.

### Method details

This was a feasibility study conducted in accordance with the Consolidated Standards of Reporting Trials statement (CONSORT) 2025: extension to randomized pilot and feasibility trials.^29^

#### Feasibility, acceptability, adherence, and tolerability assessment

The enrollment process, levels of interest, dropout rates, as well as technical and safety considerations throughout the study were monitored. The participants’ perceptions of satisfaction and motivation were analysed to determine how acceptable and effective they found the ADp.

The participants evaluated the feasibility and acceptability of the ADp using a 5-point Likert scale and an open-ended questionnaire (Supplemental Material 1) administered post-intervention, with durations ranging from 22 to 48 min. The 5-Likert scale is widely recognized for its validity and is commonly used in cultural and medical research to evaluate treatment expectations.^30^ The feasibility criteria and benchmarks were based on previous studies and best practice guidelines for dance interventions,^31^^,^^32^ and the questionnaire was adapted from previously validated instruments assessing dance interventions in PD.^9^^,^^24^^,^^26^ The instrument was designed to capture participants’ perspectives in a pragmatic manner, consistent with recommendations for early-phase trials.

The questionnaire was divided into three parts

1) Part I: Participant Satisfaction and Benefits Perception: This section aimed to assess the participants’ satisfaction levels and perceptions about the effects of the ADp. It included items addressing both motor and non-motor symptom improvements, comfort, well-being, enjoyment, treatment credibility and expectation, perceived benefits of the ADp on Parkinson’s symptoms, satisfaction with the environment (dance studio), and perceived instructor qualifications. Responses were measured using a 5-point Likert scale, with anchors ranging from 1 (“strongly disagree/not at all/very dissatisfied”) to 5 (“strongly agree/extremely/very satisfied”).
2) Part II: Perceived Difficulty in Performing ADp Movements: This section evaluated how participants perceived the level of difficulty when performing specific ADp movements, such as spinal flexion and extension, lateral steps, turns, arm-leg coordination, and improvisational tasks. Each item was scored on a 10-point scale, where 1 indicated “very easy” and 10 corresponded to “extremely difficult.” For analysis, individual item scores were averaged to generate an overall difficulty index for each participant.
3) Part III: Open-ended Qualitative Feedback: The final section allowed participants to provide open-ended responses, offering space for complaints, suggestions, compliments, and additional reflections on the intervention. The participants could describe perceived benefits, suggest improvements, and express their willingness to continue participating in future sessions of the ADp.

All the participants who completed the ADp filled the questionnaire to gather evidence, either supporting or challenging our conclusions regarding the categorization of the intervention as ‘accepted,’ ‘modified,’ or ‘rejected.’

Adherence was evaluated based on class attendance and responses to questions about participants' interest in continuing to practice the techniques of ADp after completing the program. An adherence rate of ≥70% was considered high, while an attrition rate of ≤15% was deemed acceptable.^9^^,^^24^

Tolerability was defined as the ability to endure the intervention and execute the movements without dropping out or failing to complete the protocol. This was further assessed by tracking the occurrence of severe adverse events.^26^

The participants’ safety was a priority throughout the program. Specific measures were implemented and continuously monitored to ensure a secure environment. A multidisciplinary support team was present during the ADp classes, closely observing activities and intervening when necessary to adapt and/or modify activities to the participants’ individual needs.

The questionnaire was administered individually (1:1) by a single trained evaluator to ensure standardization and consistency across participants. If participants expressed any uncertainty, the evaluator restated the question for clarity and provided verbal reinforcement of the instructions when necessary to support participants’ understanding.

#### Supplemental material 1. 5-point Likert scale and an open-ended questionnaire

##### Part I – satisfaction and perception (5-point Likert scale)

Participants were asked to rate each statement based on their level of agreement or satisfaction, using a 5-point Likert scale:

1 Strongly disagree/Not at all/Very dissatisfied
2 Disagree/Slightly/Dissatisfied
3 Neutral/Moderately
4 Agree/Considerably/Satisfied
5 Strongly agree/Extremely/Very satisfied

Statements:

1. What is your level of satisfaction with the activities carried out in the Amazonian Dance Project?
2. To what extent do you believe that regular practice of Amazonian dances can contribute to improving your non-motor symptoms (e.g., memory, language)?
3. To what extent do you believe that regular practice of Amazonian dances can contribute to improving your motor symptoms (e.g., gait, rigidity, slowness)?
4. To what extent do you believe that practicing Amazonian dances can promote a sense of well-being and joy?
5. How comfortable did you feel participating in the Amazonian dance sessions within the Dance for Parkinson’s Project?
6. How motivated did you feel to participate in the Amazonian dance sessions within the Dance for Parkinson’s Project?
7. Did you feel afraid of falling during the Amazonian dance activities?
8. What is your level of satisfaction with the physical space/dance studio provided for the Amazonian dance sessions in our project?
9. How qualified do you consider the teachers and instructors of Amazonian dances in the Dance for Parkinson’s Project?

##### Part II – movement difficulty (numeric rating scale)

Participants were asked to rate the difficulty of performing specific movements using a numeric scale from 1 (very easy) to 10 (extremely difficult).

Movements Evaluated:

Flexing and extending the spine

Lateral steps

Turning around (rotational movements)

Coordinating arms and legs simultaneously

Improvising movements

Transferring body weight from one side to another

##### Part III – Open-ended questions

Participants were encouraged to provide qualitative feedback, suggestions, or comments regarding their experiences in the program.

Questions:

1. Did you experience any prolonged adverse effects (e.g., dizziness, muscle soreness, or others)?
2. What was the most important thing you learned from participating in this program?
3. Do you have any recommendations for the research team regarding what could be improved in designing future studies?

**Note:** This questionnaire was designed specifically for this feasibility study to assess participant satisfaction, perceived movement difficulty, and qualitative feedback regarding the Amazonian Dance Protocol (ADP) for individuals with Parkinson’s disease.

#### Demographic data and clinical assessment

Participants’ demographic and clinical data were assessed through structured questionnaires administered pre-intervention. Demographic information includes age, sex, race/ethnicity, marital status, and education level. Additionally, the time since the diagnosis of PD was recorded, an important clinical variable that may affect both the symptoms severity and adherence to treatment. The dosage of Prolopa was also recorded to assess medication usage. Global cognition, previous dance experience, and fear of falling assessment were used for sample characterization.

The Goldsmith’s Dance Sophistication Index (Gold-DSI) was used to assess the participants’ level of dance experience. This questionnaire provides a brief, standardized, and continuous evaluation of doing, watching, and knowing about dance,^33^ and has already been validated for the Brazilian population.^34^

The MoCA (Montreal Cognitive Assessment) was used to assess global cognition. In Brazil, the interpretation of MoCA scores varies according to educational level and group differences.^21^ The MoCA cutoff score to differentiate cognitively unimpaired (CU) from people with dementia is 15. To distinguish cognitively unimpaired (CU) from people mild cognitive impairment-range (MCI-range), the cutoff score is 19. Furthermore, the MoCA cutoff score for identifying MCI varies according to individuals' educational levels, reflecting the importance of tailoring assessments to educational contexts. For individuals with no formal education, the cutoff score is 11.5. Those with 1–4 years of education have a cutoff score of 18. Individuals with 5–8 years of education have a cutoff score of 19.5, as do those with 9–11 years of schooling. Finally, for individuals with 12 or more years of education, the cutoff score is 22. These values underscore the need to consider educational context in interpreting results, ensuring greater accuracy in diagnosing cognitive impairment across diverse populations.^21^

The Falls Efficacy Scale - International (FES-I) questionnaire was used to assess fear of falling. The scale is rated on a four-point system, with total scores ranging from 16 (not concerned) to 64 points (very concerned). Thus, the higher the score, the greater the individual’s concern about the possibility of falls.^35^ Thus, a score of 23 or higher would be associated with occasional falls, while a score above 31 would indicate an association with recurrent falls.^36^

#### Amazonian dance intervention

The full study protocol has been described previously.^20^ The Amazonian Dance protocol (ADp) comprised 24 sessions conducted twice a week, each lasting 60 min, in a setting designed for safe and effective practice. The space was spacious, well-ventilated, and level, equipped with appropriate facilities, including non-slip flooring, sturdy chairs, support bars, mirrors, and a high-quality sound system. The sessions were led by qualified dance professionals with backgrounds in dance and physical education, specialized in dance, who were also experienced dancers and had experience teaching PwP.

Instructors who delivered the ADp protocol followed a standardized manual including a curated playlist, structured lesson plans, and instructional videos (see Supplementary Material – Mendeley Data). They received specific training, and protocol fidelity and consistency across instructors were ensured through direct observation, standardized checklists, and regular supervisory team meetings.

Figure 1 illustrates the structure of the ADp sessions.

### Quantification and statistical analysis

Descriptive (mean and standard deviation) and percentage data was used to present the participants’ demographic and clinical characteristics, and feasibility and acceptability benchmark goals and evaluation.

Adherence to the ADp was evaluated by monitoring participation rates, including the total number of completed sessions and the frequency of attendance. To be considered sufficiently adherent, the participants had to attend at least 75% of the scheduled sessions. Additionally, potential barriers to participation, such as transportation issues, physical limitations, and personal constraints, were identified to assess feasibility and propose strategies to improve adherence.

The qualitative data, accessed via open-ended questions, was analysed using a collaborative and iterative process involving the vdevelopment of an analytical framework and a systematic approach to data analysis.^37^ A hybrid coding procedure was employed, ensuring anonymity and confidentiality for all participants. All 16 semi-structured interviews were thoroughly examined to comprehensively capture participants' perceptions and experiences. This approach provided a transparent audit trail, enhancing the reliability and trustworthiness of the findings. Only initials will be used throughout the article to maintain participant anonymity, following ethical research guidelines.

### Additional resources

The ADp was developed in accordance with the Template for Intervention Description and Replication (TIDieR) reporting guidelines^38^ and was prospectively registered at ClinicalTrials.gov (ID: NCT06967493).^38^

This study was supported by the Alzheimer’s Association and the Global Brain Health Institute (GBHI) under grant GBHI ALZ UK-24-1068625, and by the Coordenação de Aperfeiçoamento de Pessoal de Nível Superior – Brasil (CAPES) – Finance Code 001. The authors declare no conflicts of interest.
